# Using Magnetic Resonance for Predicting Femoral Strength: Added Value with respect to Bone Densitometry

**DOI:** 10.1155/2015/801518

**Published:** 2015-08-27

**Authors:** Olivia Louis, Yves Fierens, Maria Strantza, Robert Luypaert, Johan de Mey, Erik Cattrysse

**Affiliations:** ^1^Department of Radiology, UZ Brussel, Vrije Universiteit Brussel, Laarbeeklaan 101, 1090 Brussels, Belgium; ^2^Department of Mechanics of Materials and Constructions, Vrije Universiteit Brussel, Pleinlaan 2, 1040 Brussels, Belgium; ^3^Department of Experimental Anatomy, Vrije Universiteit Brussel, Laarbeeklaan 103, 1090 Brussels, Belgium

## Abstract

*Background and Purpose*. To evaluate the added value of MRI with respect to peripheral quantitative computed tomography (pQCT) and dual energy X-ray absorptiometry (DXA) for predicting femoral strength. *Material and Methods*. Bone mineral density (BMD) of eighteen femur specimens was assessed with pQCT, DXA, and MRI (using ultrashort echo times (UTE) and the MicroView software). Subsequently biomechanical testing was performed to assess failure load. Simple and multiple linear regression were used with failure load as the dependent variable. *Results*. Simple linear regression allowed a prediction of failure load with either pQCT, DXA, or MRI in an *r* ^2^ range of 0.41–0.48. Multiple linear regression with pQCT, DXA, and MRI yielded the best prediction (*r* ^2^ = 0.68). *Conclusions*. The accuracy of MRI, using UTE and MicroView software, to predict femoral strength compares well with that of pQCT or DXA. Furthermore, the inclusion of MRI in a multiple-regression model yields the best prediction.

## 1. Introduction

Hip fractures represent one of the most deleterious consequences of osteoporosis. Bone strength is routinely evaluated using bone mineral density (BMD), measured either with quantitative computed tomography (QCT), as a volumetric density, or with dual-energy X-ray absorptiometry (DXA), as an areal density. These techniques provide accurate measures for BMD [1, 2], but they have the disadvantage of using X-rays. The ability of quantitative computed tomography (QCT) and/or DXA to predict femoral strength as assessed using biomechanical testing has been evaluated in some previous studies [3–6]. The conclusion was that adding some texture parameter obtained with high-resolution digital X-ray or some geometrical parameter derived from QCT or DXA improved the prediction obtained from BMD. Another technique, magnetic resonance imaging (MRI), has also been found to be suitable for assessing bone. Several groups have shown that geometrical variables can be assessed using MRI at the level of the femur or the tibia [7–9]. In a clinical setting, MRI has the advantage of examining the patient without ionizing irradiation but also the disadvantage of not measuring BMD.

In the present study, our objective was to compare the ability of the BMD measures derived from peripheral QCT (pQCT), DXA, and MRI to predict femoral strength. In particular, we wanted to evaluate the added value of MRI with respect to classical bone densitometry techniques. For this purpose, we have examined excised femurs with pQCT, DXA, and MRI, subsequently performing biomechanical testing in order to obtain a neck fracture and to assess the failure load. The MRI consisted of an ultrashort-echo-time acquisition. After segmentation of the resulting images, BMD values were derived using the MicroView Advanced Bone Analysis Applications software (GE Healthcare, Illinois, USA).

## 2. Materials and Methods

### 2.1. Specimens

This study was performed on 18 femur specimens excised from 18 cadavers. All cadavers had been donated through a body donation program of our university and had been preserved using the injection of a formalin solution in the vessels. Eleven of the specimens originated from women and seven from men. The age of the subjects ranged from 73 to 97 years. The main causes of death were heart failure and cerebrovascular insult. Malignancy, chronic infection, diabetes, or treatment susceptible to interfere with bone metabolism was not present in any of the cases.

### 2.2. Peripheral Quantitative Computed Tomography

Single-energy pQCT was performed using a Stratec XCT 2000 device (Stratec, Pforzheim, Germany) with a 37 keV X-ray tube as source of radiation. All the examinations were performed by the same experienced radiologist (OL). In each specimen, four slices (2 mm thick) at the level of the femoral neck and situated at, respectively, 1%, 2%, 3%, and 4% of the whole femoral length below the upper limit of the neck were studied. Trabecular and cortical bone were analyzed separately. The threshold used to define cortical bone was fixed at a linear attenuation coefficient of 0.93 cm^−1^ to minimize partial volume effect. The total bone mineral density of the first, second, third, and fourth slice (pQCT-BMD_1_, pQCT-BMD_2_, pQCT-BMD_3_, and pQCT-BMD_4_) was reported. All densities were expressed as milligrams hydroxyapatite of calcium per millilitre.

### 2.3. Dual-Energy X-Ray Absorptiometry

Dual-energy X-ray absorptiometry was performed using a Hologic Discovery apparatus (Waltham, Massachusetts, USA), with an X-ray source pulsed alternately at 100 and 140 kVp (effective beam energies 43 and 110 KV). All the examinations were performed by the same experienced radiologist (OL). The femurs were scanned while being immersed in a 16 cm water bath. Image acquisition and analysis were done following the recommendations of the manufacturer. Areal BMD (mg/cm^2^) was assessed. Here, the bone mineral density of the total hip (DXA-BMD_tot_), and at the level of the neck (DXA-BMD_ne_), the cortical thickness at the level of the neck, expressed as mm (DXA-CoT_ne_), and the neck shaft angle, expressed as degrees (DXA-NSA), were reported.

### 2.4. Magnetic Resonance Imaging

All femurs were scanned using a 3 Tesla Philips Achieva TX MRI system (Best, Netherlands), using a standard 8-channel knee coil, a 3D radial acquisition technique, and ultrashort echo times (UTE). All the examinations were performed by the same physicist (YF). UTE sequences allow estimation of bone *R*2^∗^ and permit subsequent segmentation of the bone. This technique was previously used for attenuation correction methods in PET-MRI systems [10]. Our sequence combined two echo times (0.14 ms and 2.6 ms). Repetition time (TR) was 20 ms. Voxel size was 0.4 × 0.4 × 0.4 mm. The angular density of the radial profiles was 200%, in order to prevent undersampling of the boundary of k-space due to the radial acquisition trajectories. Starting from the resulting images, 2 mm thick multiplanar reconstructions corresponding to the locations of the four pQCT slices were made using the manufacturer's software. The corresponding *R*2^∗^ maps were calculated according to

(1) $$\begin{array}{c} R2^{*}=\frac{\ln\left(S_{\text{short}}\right)-\ln\left(S_{\text{long}}\right)}{{\text{TE}}_{\text{long}}-{\text{TE}}_{\text{short}}}, \end{array}$$

where TE_long_ and TE_short_ are the echo times and *S*_short_ and *S*_long_ are the corresponding signals.

Using thresholding, segmentation into bone, air, and tissue was obtained. Air was masked using a signal threshold on the short echo images. The threshold between tissue and bone (*R*2^∗^_tresh_ = 0.3 MHz) was determined on the basis of a single femur and applied for all specimens. The segmented images were mapped into pseudo-computed-tomography images using standard Hounsfield units for each tissue (−1000 for air, 0 for tissue, and 1000 for bone). Finally, this pseudo-computed-tomography image was analyzed using the MicroView Advanced Bone Analysis Applications (GE Healthcare, Illinois, USA) [11] to obtain BMD values for all four slice positions. The reported variables were MRI-BMD_1_, MRI-BMD_2_, MRI-BMD_3_, and MRI-BMD_4_, corresponding to the total bone mineral density assessed, respectively, at the first, second, third, and fourth slice. The densities were expressed as milligrams hydroxyapatite of calcium per milliliter.

### 2.5. Biomechanical Testing

**Figure 1:**
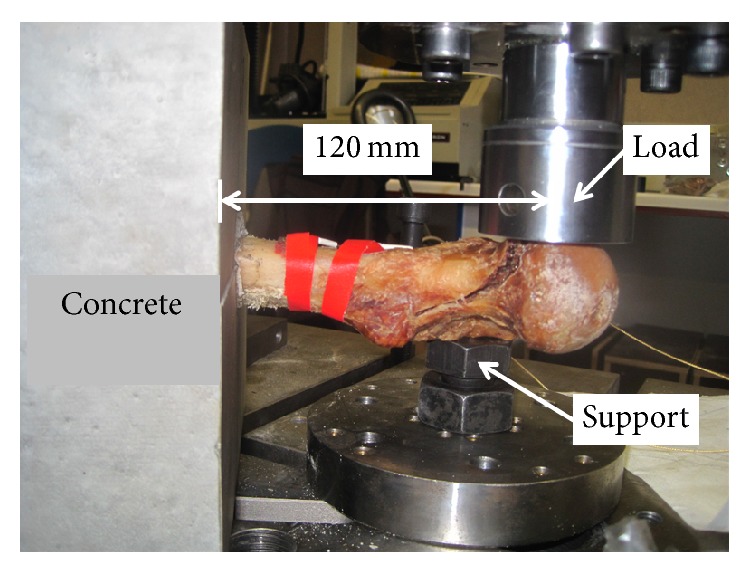
Biomechanical test of an excised femur partially encased in concrete.

First, an ultrasonic study was performed on some of the femur specimens in order to evaluate the elastic wave propagation in human femur tissues [12]. Next, the femur specimens were prepared for biomechanical testing by encasing the three distal quarters of the femoral diaphysis in concrete (Figure 1). The distance between the head of the femur specimen and the concrete fixation was 120 mm. In order to avoid the fracture at the fixation point and to mimic a neck fracture, a support was provided at the level of the main body using a metal bolt. The geometry of the setup resulted in a combination of bending and torsion. An Instron 5885 (Buckinghamshire, UK) machine was used, with 10 kN load cell and a constant rate of displacement of 2 mm/min. All the examinations were performed by the same experienced physicist (MS). The testing machine had been validated in our laboratory, as recommended by Turner and Burr [13], using plastics material standards, and had been used previously to assess the compressive strength of peripheral bone specimens [14]. Load-displacement curves were recorded during test and the failure load, expressed in Newton (N), was reported.

### 2.6. Statistical Analysis

The collected data were reported as mean (standard deviation (SD)). Linear regression analysis was performed using SPSS, version 20 (IBM Corp, Armonk, NY). Results were reported as coefficients of determination (squared correlation coefficients) and as prediction equations (load as dependent variable).

## 3. Results

**Figure 2:**
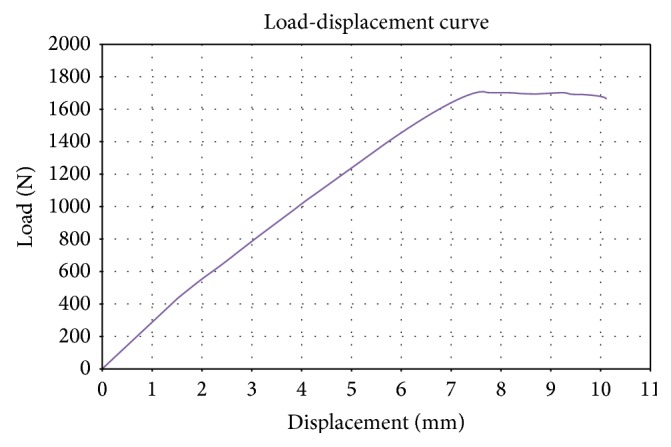
Biomechanical testing: load (N) plotted against displacement (mm).

Mean (SD) values of the variables obtained from pQCT, DXA, and MRI, as well as the failure load, are listed in Table 1. A specimen, partially encased in concrete, as submitted to the test, is shown in Figure 1. As expected, a neck fracture occurred in all specimens. A typical load-displacement curve is shown in Figure 2.

Simple linear regression analysis showed that the best prediction of failure load was obtained with the density measured at the level of the second slice for pQCT (*r* ^2^ = 0.48, *P* = 0.002), the total density for DXA (*r* ^2^ = 0.43, *P* = 0.003), and the total density assessed at the level of the first slice for MRI (*r* ^2^ = 0.41, *P* = 0.004). All coefficients of determination are listed in Table 2.

In a second step, we tested multiple-regression models, using the variables ranked best in the simple linear regression analysis, namely, pQCT-BMD_2_, DXA-BMD_tot_, and MRI-BMD_1_. The stepwise and reverse-stepwise procedures yielded the same best models.

Including only pQCT and DXA, the best prediction model was 

(2) Load=−1417.9+8.36×pQCT-BMD2 +1.96×DXA-BMDtotr2=0.50,  P=0.006.

Adding MRI improved the prediction:

(3) Load=−1674.9+7.03×pQCT-BMD2 +(1.33×DXA-BMDtot) +4.96×MRI-BMD1r2=0.68,  P=0.001.

**Figure 3:**
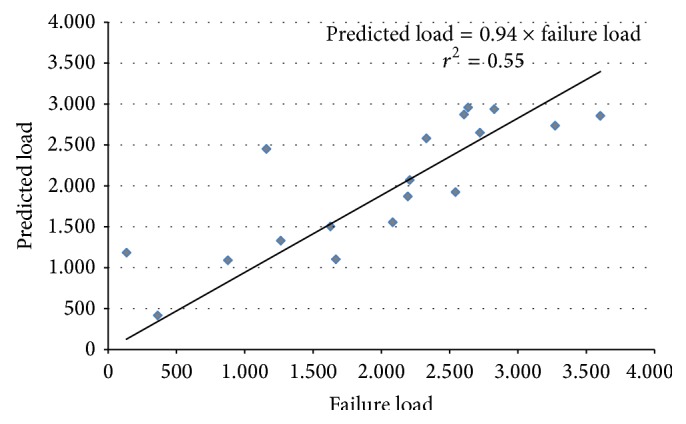
Predicted load from the multiple linear regression model plotted against the measured failure load (both expressed in N).

The agreement between the load predicted by the model and the observed failure load is illustrated in Figure 3.

## 4. Discussion

In the present study we examined 18 excised femurs consecutively with pQCT, DXA (after immersion in a water bath), and ultrashort-echo-time MRI. Subsequently, the femurs were subjected to biochemical testing. Linear regression analysis showed that the prediction of femoral strength obtained from BMD measured using MRI compared reasonably well with that obtained from BMD measured using pQCT or DXA. Furthermore, the optimal prediction was obtained with a model including one variable from each of the three methods.

Earlier studies of the relationship between conventional bone densitometry and biomechanical properties of femur specimens have examined the potential added value of bone-imaging techniques. Several authors have found that bone texture parameters, derived from using a high-resolution digital X-ray device, are as effective as the BMD generated by DXA in predicting the maximal failure load on biomechanical testing [5, 6]. Similarly, other groups have found that combining BMD measured by DXA with texture parameters allowed a better prediction of the failure load of excised femurs than that obtained with BMD alone [15, 16]. Where bone densitometry techniques are concerned, some groups focused their study on a comparison between DXA and QCT. Cheng et al. concluded that DXA and QCT had a similar ability to predict femoral strength in vitro, the best DXA parameter being the trochanteric BMD and the best QCT parameter the cortical area [3]. On the other hand, Bousson et al. concluded that QCT was a better predictor of failure load than DXA [4] in case of cervical fracture of the proximal femur. Until now, few studies have examined the ability of MRI to predict femoral strength and all these studies have focused on geometrical parameters. Manske et al. reported that the cortical area of the femoral neck, assessed using MRI, was significantly associated with failure load, with, however, a weaker prediction than that obtained with BMD using DXA [17]. Similarly, Bae et al., studying femur cortical bone slabs and using UTE, reported significant associations between MRI variables and mechanical properties, with, however, the best *r* ^2^ being only 0.31 [18].

In the present study we chose pQCT rather than QCT because pQCT allows studying slices at different levels of the femoral neck. So doing, we obtained the best prediction of femoral strength with the density assessed at the second slice. The main originality of this study is however the segmentation of the ultrashort-echo-time MRI images and their subsequent analysis using the MicroView software, allowing us to obtain BMD values. This approach differs from that used in previous MRI bone studies, which all focused on geometrical parameters [17–19]. Using this approach we obtained a degree of prediction of failure load comparing reasonably well to that obtained with pQCT-derived or DXA-derived density. Furthermore, the addition of MRI-derived BMD to BMD derived from pQCT and DXA in a multiple linear regression model improved the coefficient of determination from 0.50 to 0.68.

Our study has strengths and limitations. To assess BMD we used DXA, the most frequently used method worldwide, but also two three-dimensional techniques, able to measure a true volumetric density. Furthermore, the specimens were obtained from subjects without any known disease interfering with bone metabolism. The main limitation of our study is the relatively small number of femurs, including both genders. In addition, we cannot exclude that the injection of a formalin solution might have some effect on the mechanical properties of the bone specimens. Finally, our results derived from the biomechanical testing cannot be extrapolated to in vivo injury characterized by complex interactions related to fall conditions, overlying soft tissue, and muscular strength.

## 5. Conclusions

The present study performed on femur specimens suggests that MRI using UTE and a subsequent analysis with the MicroView Advanced Bone Analysis Applications software is able to measure BMD. This MRI-derived BMD correlates with failure load to an extent comparing well with BMD derived from classical bone densitometry techniques (pQCT or DXA). Furthermore, when using a multiple linear regression model, the addition of MRI improves the prediction of femoral strength.

## Figures and Tables

**Table 1:** Descriptive statistics of 18 femur specimens.

	Unit	Mean	SD
Age	Year	83.9	6.5
pQCT-BMD_1_	mg/mL	250.4	58.2
pQCT-BMD_2_	mg/mL	230.2	53.6
pQCT-BMD_3_	mg/mL	228.3	45.4
pQCT-BMD_4_	mg/mL	241.2	51.6
DXA-BMD_tot_	mg/cm^2^	763.0	131.0
DXA-BMD_ne_	mg/cm^2^	613.0	123.0
DXA-CoT_ne_	mm	1.3	0.3
DXA-NSA	Degree	131.8	4.5
MRI-BMD_1_	mg/mL	212.4	86.5
MRI-BMD_2_	mg/mL	200.1	79.9
MRI-BMD_3_	mg/mL	199.9	76.3
MRI-BMD_4_	mg/mL	180.2	71.5
Load	N	2005.8	957.1

pQCT-BMD_1_, pQCT-BMD_2_, pQCT-BMD_3_, and pQCT-BMD_4_: total bone mineral density measured with pQCT, respectively, at the first, second, third, and fourth slice.

DXA-BMD_tot_, DXA-BMd_ne_, DXA-CoT_ne_, and DXA-NSA: bone mineral density of the total hip and at the level of the neck, cortical thickness at the level of the neck and neck shaft angle, measured with DXA.

MRI-BMD_1_, MRI-BMD_2_, MRI-BMD_3_, and MRI-BMD_4_: total bone mineral density measured with MRI, respectively, at the first, second, third, and fourth slice.

**Table 2:** Simple linear regression analysis (with load as dependent variable).

Measure	*r* ^2^	*P*
pQCT-BMD_1_	0.36	0.009
pQCT-BMD_2_	0.48	0.002
pQCT-BMD_3_	0.42	0.004
pQCT-BMD_4_	0.15	0.110

DXA-BMD_tot_	0.43	0.003
DXA-BMD_ne_	0.41	0.004
DXA-CoT_ne_	0.41	0.004
DXA-NSA	0.09	0.269

MRI-BMD_1_	0.41	0.004
MRI-BMD_2_	0.32	0.015
MRI-BMD_3_	0.08	0.248
MRI-BMD_4_	0.17	0.084

The values in the table are coefficients of determination (*r* ^2^) and significance levels (*P* values).

Same abbreviations as in Table 1.
